# Neonatal Emergency Transport Organisation and Activities in Italy—The Nationwide 2023 Survey by the Neonatal Transport Study Group of the Italian Society of Neonatology

**DOI:** 10.3390/children12020162

**Published:** 2025-01-29

**Authors:** Carlo Bellini, Maurizio Gente, Diego Minghetti, on behalf of the Neonatal Transport Study Group of the Italian Society of Neonatology

**Affiliations:** 1 Neonatal Emergency Transport Service, Neonatal Intensive Care Unit, IRCCS Istituto Giannina Gaslini, 16147 Genoa, Italy; diegominghetti@gaslini.org; 2 Neonatal Emergency Transport Service, Lazio Regional Emergency 118 Department, 00149 Rome, Italy; maurizio.gente@libero.it

**Keywords:** infant, newborn, intensive care, neonatal transport, perinatal care, regional medical programmes, health services accessibility

## Abstract

**Background**: The regionalisation of perinatal care emphasises the importance of transferring high-risk pregnancies “in utero” to minimise risks; yet, neonatal inter-facility transport remains necessary. Neonatal Emergency Transport Services (NETSs) play a crucial role in reducing transportation risks, especially for very preterm infants. Italy’s healthcare system, which is decentralised in nature, leads to variations in NETS organisation and resources across the country, resulting in disparities in access and quality of care. **Methods**: A questionnaire regarding neonatal transfer practices and NETS activity was sent to the 55 NETSs operating in twenty Italian regions. Demographic data were obtained from the Italian National Statistical Institute (ISTAT). **Results**: Survey Overview. A 2022 national survey by the Italian Society of Neonatology aimed to assess the status of NETS in Italy, achieving a 100% response rate from the 55 NETS. The 2022 data highlighted the transport of 6494 neonatal, of which 92% were primary transports (transferred to higher-level care) and 553 were back-transports (returning stabilised neonates to lower-level care). Subgroup analysis identified 544 transports of neonates born at 30–34 weeks of gestation and 305 transports of neonates born at under 30 weeks of gestation. This was shown to have regional variability. NETS coverage: 18 regions have full NETS coverage. Sicily offers partial coverage. Sardinia, despite an approved plan, lacks an operational NETS. Operational models: all NETS provide a 24/7 service; 50 NETSs rely on an on-call basis using NICU staff for transport. Only five NETS have dedicated teams exclusively for neonatal transport. This decentralisation results in heterogeneity in service availability, access, and quality. **Conclusions**: This study highlights that although differences still exist, the NETS in Italy is adequately structured and effective. The presence of NETS operating with limited transport volumes puts a strain on maintaining skilled staff and cost-effective operations. Regional disparities: inequities in NETS access (e.g., in Sicily and Sardinia regions) underline the need to improve regional collaboration. While Italy has made progress in organising NETS, regional discrepancies persist in access and service quality, reflecting the decentralised nature of its healthcare system.

## 1. Introduction

The regionalisation of perinatal care recommends that high-risk pregnancies should be managed in tertiary hospitals. When transfer is necessary, maternal transfer ’in utero’ is preferred to reduce risks to both the mother and newborn [1,2]. However, although this organisational model is widely regarded as the best approach, it is not always possible to anticipate or prevent all complications related to pregnancy and childbirth. As a result, a proportion of newborns will inevitably require access to neonatal transport services after birth [1,2,3,4,5]. To ensure that these newborns receive the required level of care, a well-organised Neonatal Emergency Transport Service (NETS) must be established as part of an effective regional perinatal network [5,6]. In Italy, this systematic regionalisation of perinatal care began in the 1990s, leading to a progressive increase in the number of active NETSs and continuous improvements in service quality and coverage [5,6,7].

The current study aims to examine the protocols, practices, and challenges of neonatal transport in Italy and to provide an overview of the organisation of NETSs and their role within the framework of perinatal care. Our focus is on the critical function of a well-organised NETS within regional perinatal networks to reduce the risks associated with the transport of critically ill neonates, particularly extremely preterm infants.

Due to the decentralised nature of healthcare in Italy, each region has the authority to set its own health policy, resulting in regional variations in healthcare resources, organisational models, and outcomes.

The Italian territorial division into 20 regions is not homogeneous; there are regions with a very large territory containing large and important cities (e.g., Lombardy with Milan or Lazio with Rome), while other regions have a limited territory with cities of no more than 50–100,000 inhabitants. The distribution of hospitals, regardless of the level of care they provide, is also uneven, with some regions having a large number of major hospitals and others having only first-level hospitals. According to 2022 data [8,9], the total number of maternity units nationwide was 395, of which 137 had at least 1000 deliveries per year. On the other hand, 7.5 per cent of births took place in facilities with less than 500 annual deliveries. The neonatal intensive care unit (NICU) was present in 120 of the 395 birth centres; 91 NICUs were present at the 137 birth centres with at least 1000 annual deliveries. Non-intensive neonatal units were present at 228 birth units, of which 112 had more than 1000 annual deliveries. The total number of live births registered in 2022 was 392,598. The neonatal mortality rate in 2022 was 2.40 for stillbirths per 1000 live births. Healthcare in Italy is guaranteed by the state for all Italian citizens. In the specific case of perinatal care, every type of intervention is completely free of charge, starting with pregnancy, any special antenatal examinations that may be required, childbirth, intensive neonatal care, if necessary, with no time limit on hospitalisation and subsequent follow-up if necessary, and of course neonatal transport if necessary. In the case of foreign nationals, some of whom are undocumented, pregnancy, childbirth and postnatal care, as well as all necessary medical interventions, including transport, are considered lifesaving and, therefore, completely free. Regional autonomy can lead to discrepancies in the quality of neonatal transport and the overall quality of care, as highlighted by performance indicators from international organisations such as the Organisation for Economic Co-operation and Development (OECD) and the European Observatory on Health Systems and Policies [10,11].

The study, conducted in 2023, presents data from a national survey on NETS activities in 2022. This survey was organised by the Neonatal Transport Study Group of the Italian Society of Neonatology (SIN), with the support of the Italian Ministry of Health, and provides insight into the current status and effectiveness of neonatal transport services in Italy.

## 2. Materials and Methods

A survey was conducted in 2023 to evaluate the operational status of the Italian NETS system. This survey was organised by the Neonatal Transport Study Group of the Italian Society of Neonatology (SIN), which developed a questionnaire to ensure a high response rate and facilitate efficient data collection. The multiple-choice questionnaire consisted of 20 items focusing on the structure, operation, and activities of the NETS for the year 2022. The survey aimed to provide a comprehensive picture of the organisation, scope, coverage and activities of the NETS in Italy, specifically excluding questions on the medical outcomes of transferred newborns.

### 2.1. Survey Design and Content

The survey addressed several key areas of NETS activity, which are outlined below.

### 2.2. Annual Volume of NETS Activities

The number of transports, including primary and return transports, was determined.

The number of neonates transported at ≤28 weeks gestational age (GA) was determined.

The number of transports with neonates older than 28 days or older than 40 weeks with corrected GA for preterm neonates was determined.

### 2.3. Organisational Structure

The number and type of unit teams, distinguishing between dedicated teams and ‘on-call’ services, were determined.

The term ‘dedicated’ refers to a service whose medical staff were originally recruited from the NICU staff but who are now separated from the NICU and only provide transport services. The term ‘on-call’, on the other hand, refers to a service whose medical staff work both in the NICU and in transport and have an established rota.

### 2.4. Quality Assurance and Training

The evaluation of quality policies, training programmes, and the average time per transport were determined.

### 2.5. Transport Modalities

The types of ambulances used the availability of helicopters and options for fixed-wing aircraft for air transport were determined.

### 2.6. Approval and Dissemination

The SIN Institutional Review Board approved the survey protocol. The survey instructions, including the SIN’s certificate of approval, were distributed by email to each NETS director. The email contained a link to the online survey form, and consent was implied upon completion of the questionnaire. If no response was received by email, a telephone call was made to each NETS director, and a personal interview was conducted by the survey administrators (CB, MG, DM). The survey was not considered complete until 100% of the responses had been received.

### 2.7. Data Validation and Management

The survey administrators (CB, MG, DM) checked all submitted questionnaires for incomplete or incorrect responses. When missing data or inaccuracies were identified, the survey administrators contacted the respective NETS directors directly to ensure data completeness and accuracy. The final data were compiled in an electronic database. Demographic data for analysis were obtained from ISTAT, the Italian National Institute of Statistics.

### 2.8. Regional Data Reporting

Italy, with its 20 regions—5 of which have special autonomy—is organised as having a decentralised healthcare system that has been managed regionally since 1978. Because of this regional structure, data are presented on a regional basis rather than by individual NETS units.

### 2.9. Neonatal Transport Index

We calculated the Neonatal Transport Index (NTI) based on the number of transports per live birth multiplied by 100. We then calculated the NTI values for each Italian region.

## 3. Results

**Figure 1:**
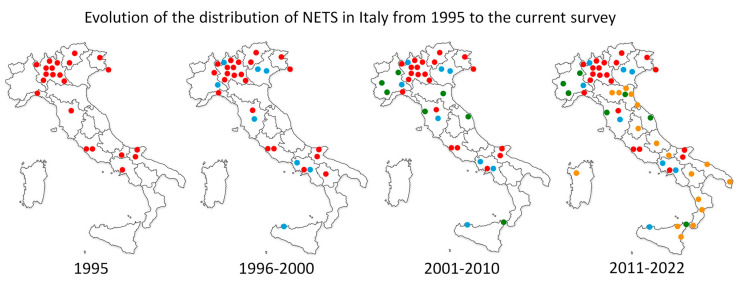
The figure shows the increase in NETSs in Italian territory starting from 1995. In the figure, the NETS operating in Turin is represented by a single dot, but in reality, it is made up of several NETSs that operate in synergy with each other. It is, therefore, a graphic trick. The different colours indicate the various NETSs that have been activated over the years, which are indicated below the map. The progression is shown in red, light blue, green, and yellow.

The survey of the NETS in Italy provides a comprehensive snapshot of service coverage and operational details across the country (Figure 1). The response rate to the questionnaire was 100% (55/55). After a single request for missing data, the 55 questionnaires were completed in full, and all were included in the analysis. The key findings are outlined below.

### 3.1. Coverage and Availability

**Figure 2:**
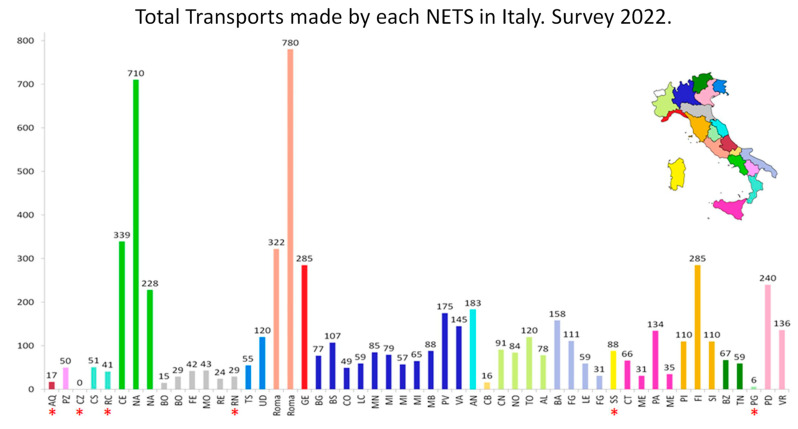
The total number of transports carried out by each individual NETS is represented. What is reported about NETS Turin in the caption of Figure 1 also applies to this figure. The asterisks refer to NETSs which have not completed a full year of activity because they were established after the start of this survey. The bars are grouped by individual regions based on colour. The small map of Italy shows the individual regions identified by the colour that corresponds to the bars in the graph. The abbreviations that appear for each single bar correspond to the official codes indicating the city that is home to the NETS. The codes correspond to those usually used in Italy, for example, for car licence plates.

**Figure 3:**
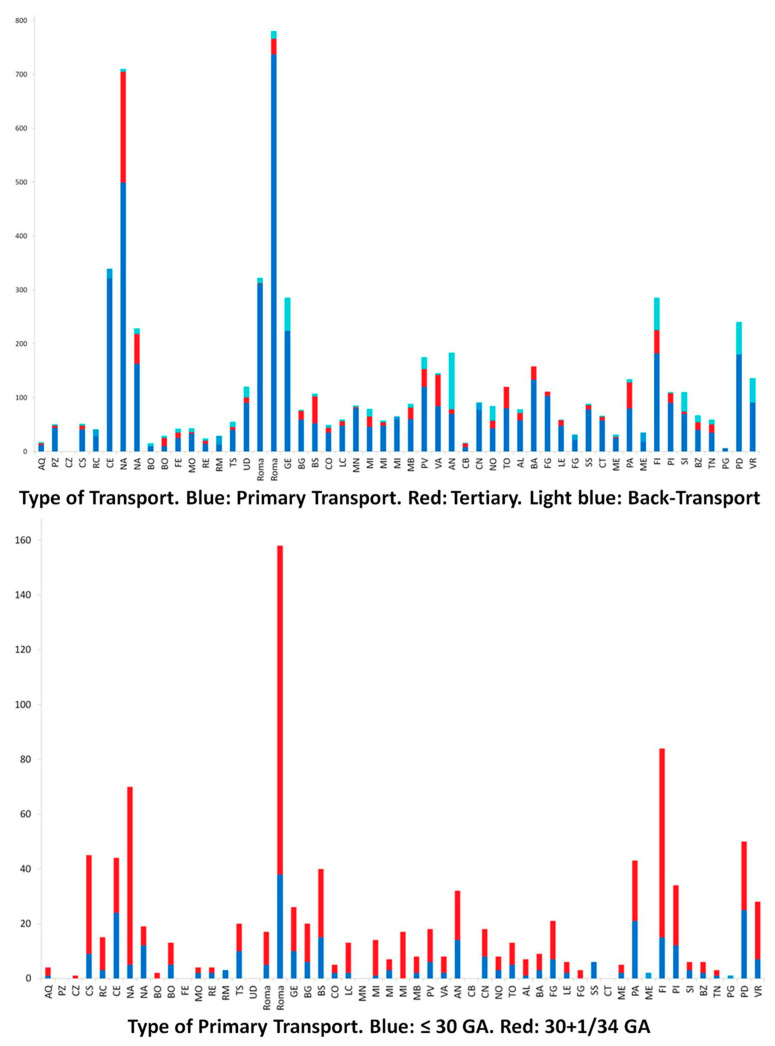
The figure shows the breakdown between the different types of transport carried out. Ga: gestational age. The colour legend of the individual bars is reported in the caption of the figures. The abbreviations that appear for each single bar correspond to the official codes indicating which city is home to the NETS. The codes correspond to those usually used in Italy, for example, for car licence plates.

**Figure 4:**
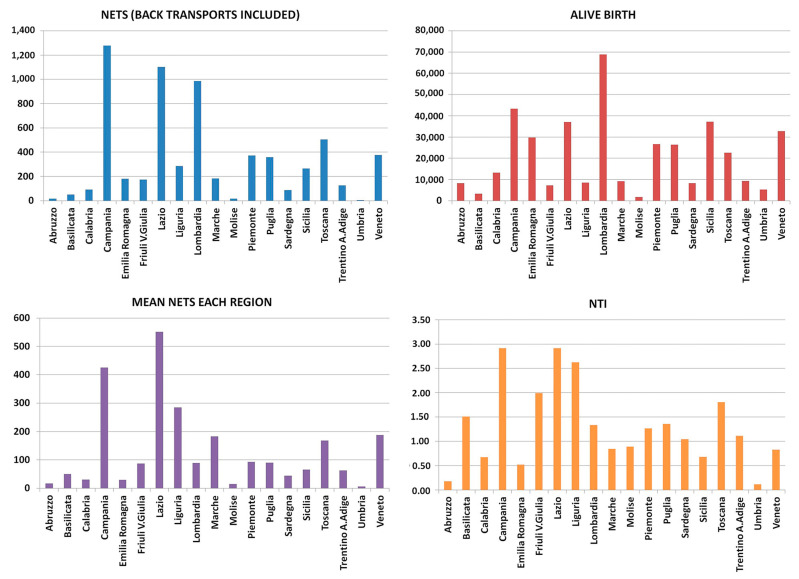
The figure shows the total number of transports divided by individual Italian region at the top left; at the top right, the number of live births for each region is given; at the bottom left, the average number of transports for each region is given, and finally, at the bottom right, the Neonatal Transport Index values (see text) for each region are given. In this figure, the different colours only serve to differentiate the topics of the graph from each other and have no other particular meaning. Each single bar indicates the result for each individual region with the name of the Italian region to which it refers.

Of the 20 Italian regions, 18 have full NETS coverage. Sicily provides partial coverage, while Sardinia does not have an active NETS despite having approved one. The Government of Sardinia has approved the establishment of the NETS, but to date, an operational resolution is not yet available and therefore, the NETS is obsolete. For this reason, it was not included in the results and is not present in Figure 2, Figure 3 and Figure 4.

All 55 NETSs provide a 24/7 service, with 50 operating on an ‘on-call’ basis using NICU staff and 5 NETS having dedicated teams for transport only.

### 3.2. Volume and Characteristics of Transport

In 2022, there were a total of 6494 neonatal transports, predominantly primary transports (5968), with fewer back-transports (553). Notable subsets include 544 transports of neonates born at 30–34 weeks and 305 of those born at less than 30 weeks (Figure 2, Figure 3 and Figure 4).

Transport times varied, with a median of 103 min (range 30–250). NETSs performed between 1 and 754 transports per year, with a median of 75 transports per NETS; only 7 NETSs performed more than 200 transports per year.

### 3.3. Specialised Equipment and Services

The availability of specialised equipment and services was mixed. Specifically, 32 NETSs could use nitric oxide [12,13,14,15,16], 24 could transport twins [17], and only 15 had phototherapy available during transport [18,19]. Only five NETS offered active cooling [20,21,22,23,24,25], and one had helium transport facilities specifically for infants.

### 3.4. Infant Transport and Clinical Conditions

Of the 55 NETS, 36 provided transport for infants older than 28 days, covering a range of clinical conditions, including respiratory distress (36 NETS), neurological problems (26), surgical needs (25), cardiac conditions (31), malformations (20), metabolic conditions (25), and trauma (2).

Age and weight criteria for infants varied between NETSs. Most accepted infants up to 3 months of age (details: age and weight limits for various age ranges: 1–3 months: 26/36; 1–6 months: 4/36; 1–12 months: 5/36; 12 months: 1/36) and 29 NETSs transported infants weighing 3–6 kg (details: weight categories of 3–6 kg: 29/36; 3–8 kg: 4/36; 3–10 kg: 1/36; 10 kg: 2/36).

### 3.5. Use of Vehicles

Dedicated ground ambulances were used by 28 NETSs [26,27], while 27 shared ambulances with local emergency services (118 in Italy, 911 in the USA or 999 in the UK).

Air transport, although limited, involved helicopters (17 NETSs) and fixed-wing aircraft (5 NETSs), with helicopter transport accounting for 0.5–15% of total annual transport, totalling 84 flights per year. Fixed-wing transport was less frequent, with a total of 15 flights [28,29,30,31].

### 3.6. Quality Assessment, Training and Education

A dedicated NETS database was available in all 55 services; specific guidelines were issued by all 52/55 NETS and, in three cases, by the ‘112 emergency service’ (118 at the time of this survey) (i.e., 911 in the USA and 999 in the UK). Regular audits were carried out by 51/55 NETSs, and in two services, this was conducted through an agreement with the 112-emergency service. Internal training and education were provided in 52/55 NETSs.

### 3.7. Neonatal Transport Index (NTI)

The NTI value for each region varied from a minimum of 0.11 to a maximum of 2.92. Of the 19 regions considered, 14 regions had NTI values below 1.5, while the remaining 5 regions exceeded this value, ranging from a minimum of 1.80 to a maximum of 2.92 [32] (Figure 4).

## 4. Discussion

A comment on the organisation and effectiveness of NETSs in Italy is provided in this section. The development and organisation of NETSs in Italy have made significant progress since their inception in the 1980s, with a particular increase since the 1990s. Similar results have been reported in Europe [33,34,35]. Despite this growth, the current status highlights a mix of successes and challenges that deserve attention for optimisation. The COVID-19 outbreak had only a partial impact on this [36,37,38,39].

### 4.1. Strengths in the Organisation of the Italian NETS

Widespread coverage, with the establishment of NETSs in 19 of the 20 Italian regions, demonstrates the strong commitment to neonatal care, with only Valle d’Aosta relying on neighbouring Piedmont for services. This indicates almost universal geographical availability.

The structured framework of the 2010 “State-Regions Conference Agreement” [40] established clear guidelines for perinatal care, including the hub-and-spoke model. This centralised approach aims to improve the quality of care through well-coordinated maternal and neonatal emergency transport systems. The ‘on-call’ model, which constitutes the majority of NETSs, uses the expertise of NICU staff to ensure high-quality care during neonatal transport.

The particular distribution of NICUs and first-level hospitals in the different Italian regions, which vary greatly in terms of population, the presence or absence of large cities, and the geographical configuration of the territory, has led to the adoption of the on-call NETS model. Staff are, therefore, employed in both NICU and neonatal transport, ensuring optimal skills and professional development. The limited number of transports per year per individual “on-call” NETS, which is common within the national activity, does not justify the activation of “dedicated” NETSs, which need to reach a much higher volume of activity in order to be functionally effective.

In addition, the fact that the staff involved in dedicated NETS activity are actually recruited within the NICUs but then move away from neonatal intensive care to transport activities makes it more difficult to maintain a high level of professional training, as the staff are highly specialised but far removed from the training activities typical of a NICU.

### 4.2. Key Challenges for NETS Implementation

Regarding the variation in regional activity levels, the data show significant variations in activity levels between regions and between individual NETSs. This imbalance undermines the cost-effectiveness and sustainability of the system. For example, many NETSs do not meet the activity thresholds established as benchmarks for operational and economic efficiency [41].

Low utilisation rates were found among ‘on-call’ NETSs, with only 2 out of 50 reaching the recommended minimum of 200 transports per year. Similarly, only one out of five “dedicated” NETSs met the standard of 600 transports per year, with two others coming close. Such under-utilisation raises concerns about maintaining the necessary skills and justifying the costs [7,41].

For economic and skills implications, the low volume of transport in some regions makes these systems less cost-effective and potentially compromises staff skills due to insufficient practical exposure. This echoes the findings of previous studies [34], which highlighted the need for at least 200 transports per year for ‘on-call’ NETSs and 500–600 for ‘dedicated’ NETSs to ensure both economic viability and clinical expertise.

The Neonatal Transport Index (NTI) evaluates the values obtained by each region and highlights the inequalities and differences between Italian regions regarding the use of NETSs. We have previously shown [32,41] that the considered effective limit for the NTI value should be less than 1.5, which means that 1.5% of newborns require NETSs. The value considered normal for the NTI comes from evaluations carried out and published by the Italian Society of Neonatology [42]. So, if we consider that 1000 physiological pregnancies are likely to produce 1000 healthy newborns, all of whom will expected to undergo safe delivery in a first-level hospital, we must expect that 10–15 newborns may present with some complications at birth, which, although not particularly serious, may be such as to warrant transfer to a third-level hospital and, therefore, the activation of the NETS. Thus, if the regionalisation of care is effectively implemented, 1–1.5 newborns in a hundred may need NETSs. Higher values would indicate either the unwarranted overuse of NETS or ineffective regionalisation of perinatal care at a regional level, which could reasonably be expected to result in a number of inappropriate neonates being born in first-level hospitals.

### 4.3. Recommendations for Improvement

The consolidation of NETS regions with a low volume of activity could consider consolidating their NETSs to ensure a higher number of transports per unit, thereby optimising resources and expertise.

Regional collaboration with increased inter-regional collaboration could address inequalities by allowing low-activity regions access to higher volume centres, ensuring a better distribution of resources.

Dedicated training and specialisation with an increased focus on dedicated NETSs with specialised staff could improve the quality of service and ensure the retention of skills, even if staff are drawn from NICUs [41].

Monitoring and policy adjustments with the regular assessment of NETS performance and volume of activity should guide evidence-based policy adjustments to improve cost-effectiveness and consistency across regions.

## 5. Conclusions

Although Italy has made remarkable progress in establishing NETSs across the country, disparities in activity levels and the under-utilisation of many systems limit the effectiveness and sustainability of the current model. Addressing these challenges requires a combination of strategic consolidation, regional coordination, and policy refinement to ensure equitable, cost-effective, and high-quality neonatal transport services across the country.

This review highlights the operational scope, variability in service provision, and regional disparities in NETSs, highlighting both the robustness and areas for development within the Italian neonatal transport system.

## Data Availability

The datasets used and/or analysed during the current study are available from the corresponding author on reasonable request. The datasets used and/or analysed during the current study data are available from the Neonatal Transport Study Group of the Italian Society of Neonatology (SIN) on reasonable request.

## List of Abbreviations

GA: gestational age; ISTAT: Italian National Statistical Institute; NETS: Neonatal Emergency Transport Services; NICU: neonatal intensive care unit; NTI: Neonatal Transport Index; OECD: Organisation for Economic Co-operation and Development; SIN: Italian Society of Neonatology.
